# A novel ‘stop-hole’ strategy to extend the fatigue life of orthodontic thermoformed retainers: an *in vitro* study

**DOI:** 10.1093/ejo/cjae061

**Published:** 2024-11-11

**Authors:** Jae Sik Son, Adam Wojcik, Joseph Noar

**Affiliations:** Department of Orthodontics, UCL Eastman Dental Institute, University College London, Rockefeller Building, 21 University Street, London WC1E 6DE, United Kingdom; Department of Mechanical Engineering, Faculty of Engineering Sciences, University College London, Roberts Engineering Building, Torrington Place, London WC1E 7JE, United Kingdom; Department of Orthodontics, UCL Eastman Dental Institute, University College London, Rockefeller Building, 21 University Street, London WC1E 6DE, United Kingdom

**Keywords:** thermoformed retainers, stop-hole, crack propagation, fatigue testing

## Abstract

**Objectives:**

This study aimed to investigate the potential to limit crack propagation in thermoformed retainers (TRs) and extend their fatigue life by placing a ‘stop-hole’.

**Methods:**

Thirty-two TRs, fabricated from 1 mm thick, round polyethylene terephthalate glycol (PETG) blanks of ICONIC^®^, underwent testing within a custom-built fatigue tester. After initial crack growth of 3 mm, the TRs were divided into three groups: a control group (C) and two experimental groups based on the stop-hole placement method. In the first experimental group, stop-holes were placed using a 1 mm round carbide bur on a slow-speed handpiece (ES), while the second group utilized a heated 1-mm-width ball-ended CPITN probe (EH). Following the placement of a stop-hole at the crack-tip, all 32 TRs resumed testing until the final crack length of 10 mm was reached.

**Results:**

Stop-hole placement significantly prolonged the delay in crack re-initiation in both experimental groups compared to the control group (*P* < .001 for both). The mean re-initiation times for the ES and EH groups were 104.50 and 129.50 min, respectively, whereas the control group was 28.94 min. Consequently, this discrepancy was reflected in the total testing times, with both experimental groups requiring significantly longer time to reach the final crack length (*P* < .001). There was no significant difference in the testing times between the two experimental groups.

**Limitations:**

This is an *in vitro* study.

**Conclusions:**

The stop-hole methodology significantly extended the fatigue life of a TR in a controlled laboratory environment.

## Introduction

All orthodontic patients face the risk of relapse, which is a complex phenomenon involving multiple factors including occlusal forces, soft tissue dynamics, periodontal conditions, and ongoing facial growth [1, 2]. Orthodontic retainers play a pivotal role in maintaining the orthodontic treatment outcome by providing resistance against such factors. It is widely acknowledged that lifelong retention is important to minimize any unwanted tooth movement post-treatment [1–4].

Thermoformed retainers (TRs) have emerged as the preferred choice among orthodontists due to their favourable characteristics [5, 6]. The rising popularity of TRs can be attributed to several factors, including greater patient acceptance, ease of fitting and fabrication, relatively low laboratory costs, and their effectiveness in maintaining dental alignment [1, 2, 7, 8]. Despite the increasing utilisation of TRs, knowledge of the governing materials science and performance of these appliances, particularly with regard to their longevity, is often proprietary. This presents a significant challenge for both orthodontists and patients, as lifelong retention is intended.

The major drawback of TRs has been their limited survival time. Studies by Sun et al. and Moslemzadeh et al. showed that TRs exhibit approximately 50 per cent shorter survival times compared to Hawley retainers, with an 8-fold higher failure rate within 6 months post-treatment [9, 10]. These findings are corroborated by a retrospective study by Jin et al., which reported a median survival time of 1529 days for Hawley retainers compared to 105 days for TRs [11]. A common reason for TR failures is the development of fatigue cracks in the incisor region, eventually leading to fracture [10–13]. The process of removing or handling a TR generates torsional forces, resulting in stress concentration and corresponding damage accumulation in the incisor region [10, 12]. The TR’s thin post-thermoforming thickness in the incisor region, coupled with its horseshoe shape, exacerbates the susceptibility to stress concentration in this area [12–15].

As no investigations have been conducted to date on minimizing crack propagation in TRs, this study aims to explore methods for reducing crack propagation by drawing insights from wider engineering practices. In engineering, studies aimed at mitigating structural replacements caused by fatigue cracks have sometimes utilized the ‘stop-hole’ technique, which involves creating a hole at the end of a growing crack-tip [16–18]. By blunting the crack, this reduces the stress concentration factor, Kt, at the crack-tip, temporarily arresting crack propagation. It relies simply on the principle that crack propagation is driven by local stress concentration, which intensifies with the sharpness of the crack-tip; thus, blunting the crack-tip effectively reduces Kt and slows crack propagation [16, 19]

This study is aimed to investigate whether applying this ‘stop-hole’ method can have a place in extending the life of TRs. As this is a novel research area in orthodontics, there is no consensus within the literature on the most optimal way of creating a stop-hole. In order to begin this line of research, we postulated that any modification of the crack-tip’s geometry to a round shape, irrespective of its method of generation would have a positive impact.

## Materials and methods

### Study design

This *in vitro* study was conducted using a purpose-built fatigue tester within a controlled laboratory setting in the Department of Mechanical Engineering, University College London. A total of 32 TRs were fabricated by a specialist orthodontic technician. Subsequent modification, testing, and data analysis of the TRs were carried out by the principal investigator, J.S.

### Sample size calculation

As the research question addressed in this study was novel and had not been previously tested, conducting a formal sample size calculation was not feasible. The total sample size (*n* = 32 TRs) was determined based on empirical experience gained from a previous study using the same fatigue apparatus [20].

### Fatigue tester

The fatigue tester was specifically developed to simulate the vertical deflection of TRs during removal from the mouth or routine handling by patients. Detailed mechanical and construction information about the tester, including the steps taken to assess its validity and reliability, can be found in the preceding study by Goh [20].

**Figure 1.**
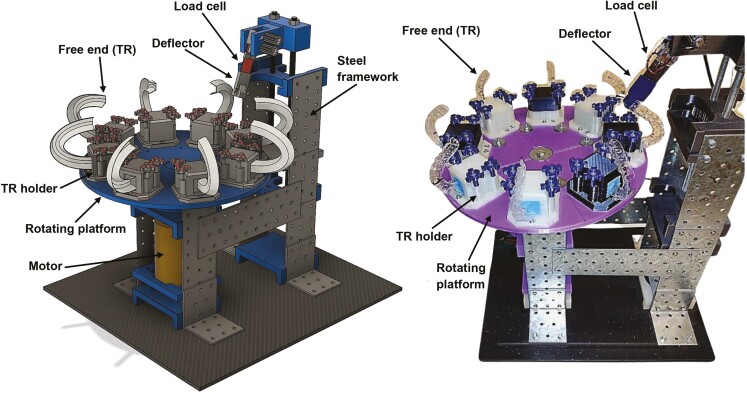
A schematic diagram and photograph of the fatigue tester.

Figure 1 illustrates the design of the fatigue tester with the TRs in place for scale. Eight TRs were loaded at a time and fatigue was tested as the rotating platform turned counterclockwise. As each TR struck the deflector, the load cell registered the generated force, which was recorded by the Picolog 6 data logging software (Pico^®^ Technology).

### TR fabrication

1-mm-thick, round polyethylene terephthalate glycol (PETG) blanks of ICONIC^®^ from DB Orthodontics were pressure-formed against a single well-aligned maxillary model. A BIOSTAR^®^ pressure-forming machine from Scheu Dental was used for the pressure-forming process. Post-thermoforming thicknesses were measured for each newly fabricated TR using a digital calliper across four predetermined points on the palatal surfaces of the upper right central incisor (UR1) and upper right lateral incisor (UR2). Only those TRs with a mean difference of 0.05 mm or less from the first TR were selected for the study to reduce scatter in the data.

### Notch placement

Cracking in materials almost always requires an ‘incubation’ stage, if no existing crack is present. To ensure consistent crack development and expedite this stage, a starter notch was utilized. A 0.5-mm-deep notch was placed using a custom-made, depth-limiting hand-held cutter fitted with a 0.017-inch rectangular carpet blade on the palatal surface between the UR1 and UR2, parallel to the long axis of the tooth.

**Figure 2.**
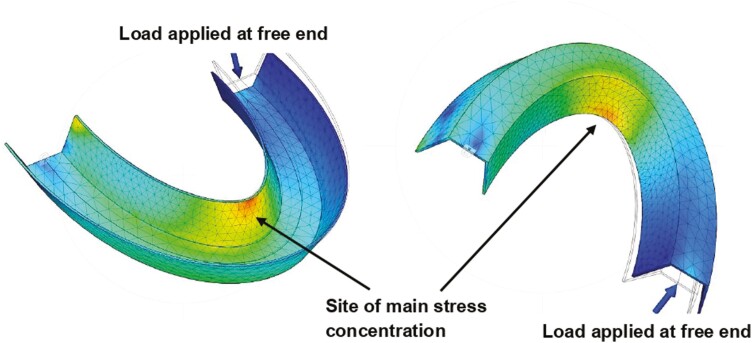
Computer simulation depicting stress distribution in a TR. The absolute values are not shown.

To model the ideal position for crack growth based on the fatigue tester setup and loading scenario, the finite element analysis package in Autodesk^®^ Fusion 360^™^ was employed. Figure 2 depicts this idealized simulation scenario highlighting the areas of stress concentration and distribution in a TR. The areas of stress concentration are visible, with the area between approximately the UR1 and UR2 on the palatal surface identified as having the highest stress concentration.

### Crack testing

All 32 TRs were selected in groups of eight using a simple randomization process and tested until a crack length of 3 mm was reached from the tip of the starter notch. Time was recorded using a computer stopwatch to the nearest 30 s. The fatigue tester was paused every 5 min to monitor for cracking and measure crack lengths under a 20× magnification microscope. As cracks propagated, the TRs became more compliant and deflected away from the applied load, thereby reducing the measured stress levels as recorded on the data logging software.

Following the formation of an initial crack line of 3 mm, the 32 TRs were evenly divided into two groups without a strict randomisation process: a control group (no. 1–16) and an experimental stop-hole group (no. 17–32). Randomization was deemed unnecessary due to the minimal variation in the times taken for the initial crack growth of 3 mm across the entire TR sample. This consistency reflected the standardized TR fabrication protocol, resulting in uniform post-thermoforming thicknesses in the region of interest throughout the sample. Furthermore, the use of a starter notch ensured that cracks were initiated from the same location, reinforcing the minimal variation observed in the TRs.

**Figure 3.**
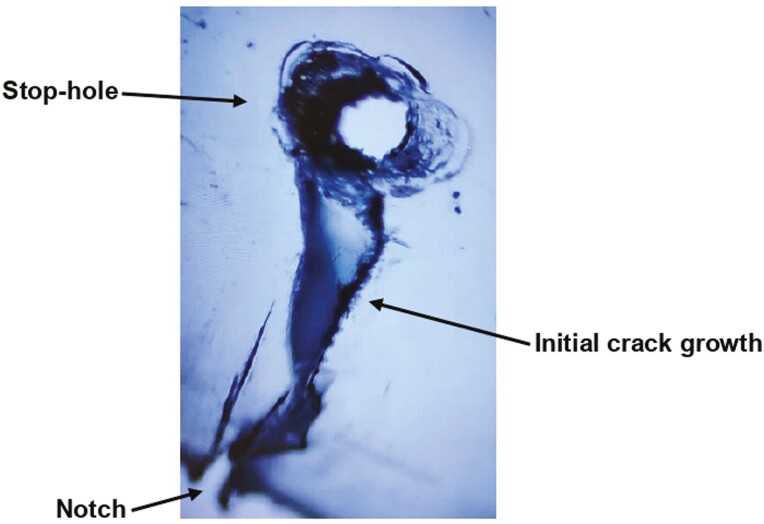
Initial crack growth and the position of a stop-hole under 20× magnification.

The 16 TRs assigned to the experimental stop-hole group were further divided into two sub-groups. Of the 16 TRs in the experimental group, eight TRs (no. 17–24) received a stop-hole with a 1 mm round carbide bur from Henry Schein^®^ (product no. H1-010) operated on a slow-speed handpiece at the end of the 3 mm crack line (Experimental group #1: ES). The other eight TRs (no. 25–32) received a stop-hole with a heated, 1-mm-width ball-ended, single-use CPITN probe from MDDI Global (Experimental group #2: EH). The position and size of the stop-holes were verified under a microscope to ensure the entire crack-tip was captured (Fig. 3). The remaining 16 TRs (control group: C) did not receive any stop-hole.

The TRs were replaced in the fatigue tester, and testing resumed. Time was once again recorded using a stopwatch on a computer, rounded to the nearest 30 s. The TRs were selected from each group in numerical order based on their labels.

**Figure 4.**
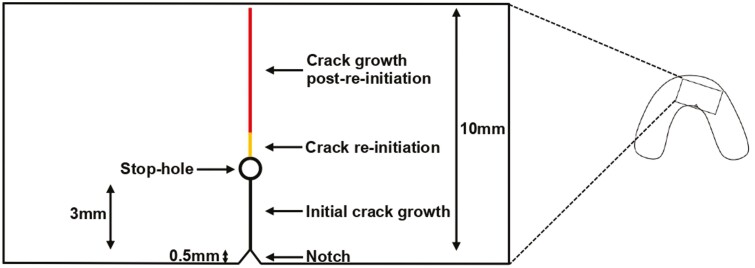
A schematic diagram of the notch placement, initial crack growth, stop-hole, and testing endpoint.

The endpoint of testing was defined as when the crack line reached a length of 10 mm from the tip of the notch, which was considered the point of failure for a TR in this experimental setting (Fig. 4). Additionally, the onset of crack re-initiation from the stop-holes was measured for the experimental groups. A 1 mm increase in crack length from the stop-holes indicated this onset. Similarly, in the control group, the time taken for the crack line to reach 4 mm from the tip of the notch was measured to facilitate comparison.

### Statistical analysis

All descriptive and analytical statistics were performed using IBM SPSS^®^ Statistics software. The times taken to reach each time point were summarized using medians, means, and standard deviations. A parametric two independent samples *t*-test was employed to compare the means between the groups. To reduce the risk of type-1 errors, a Bonferroni correction was applied to adjust the level of significance.

## Results

### Initial crack growth of 3 mm from the tip of the starter notch (0–3 mm)

**Figure 5.**
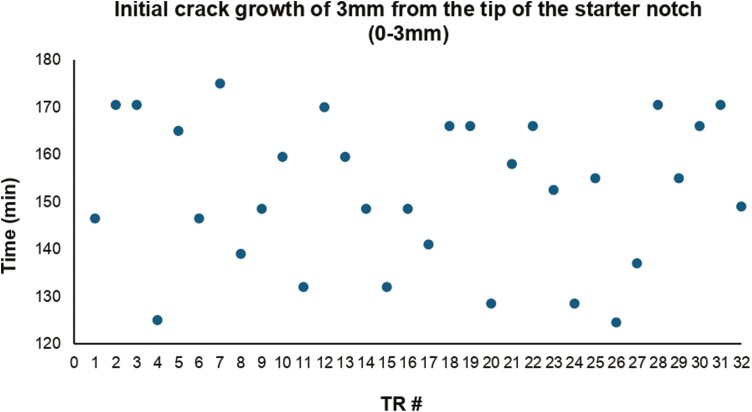
Scatter plot depicting the time taken for each TR during initial crack growth (min = minutes).

The data points on the scatter plot (Fig. 5) representing the time taken for each TR during the initial crack growth were evenly distributed, showing no evidence of any trend. A linear regression analysis (Table 1) revealed no significant relationship between the TRs during the initial crack growth (*P* = .896). Overall, there was no appreciable difference in the times taken to reach the initial crack length between the TRs.

**Table 1.** Linear regression analysis of the initial crack growth scatter plot (Fig. 5).

Test	Sig (*P*-value)	*R* square	Result
Linear regression analysis	0.896	0.001	Fail to reject null hypothesis

### Crack re-initiation

**Figure 6.**
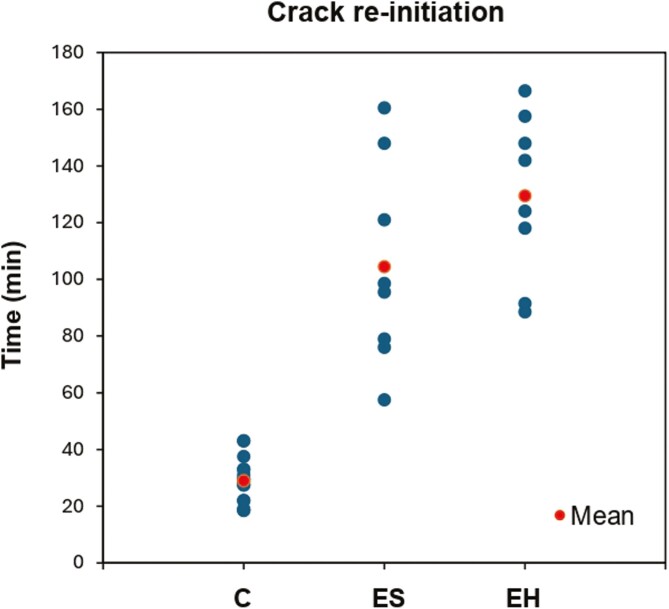
Vertical scatter plot of the crack re-initiation times across the three groups (min = minutes).

The EH group exhibited the longest mean time of 129.50 ± 29.12 min, closely followed by the ES group with 104.50 ± 36.03 min (Fig. 6). In contrast, the C group had a significantly lower mean of 28.94 ± 7.85 min. Descriptive statistics of the vertical scatter plot are summarized in Table 2. Despite the large spread in the values, there was a highly statistically significant difference in the mean times between the experimental groups and the C group (*P* < .001) (Table 3). There was no statistically significant difference between ES and EH groups (*P* = .149).

**Table 2.** Descriptive statistics of the crack re-initiation vertical scatter plot. The times should be considered within the context of the 0.5-min resolution of the experiments.

	C—Control	ES—Experimental group #1	EH—Experimental group #2
Mean	28.94	104.50	129.50
Median	29.00	97.00	133.00
Standard deviation	7.85	36.03	29.12

**Table 3.** Comparisons of the mean crack re-initiation times using a two independent samples *t*-test.

Two independent samples *t*-test	Findings					
	Sig (*P*-value)	Mean difference	Standard error	95% CI		Result
				Lower	Upper	
C vs ES	<0.001	−75.56	9.23	−94.70	−56.40	Reject null hypothesis
C vs EH	<0.001	−100.56	7.64	−116.42	−84.70	Reject null hypothesis
ES vs EH	0.149	−25.00	16.38	−60.13	10.13	Fail to reject null hypothesis

### Crack growth post-re-initiation

**Figure 7.**
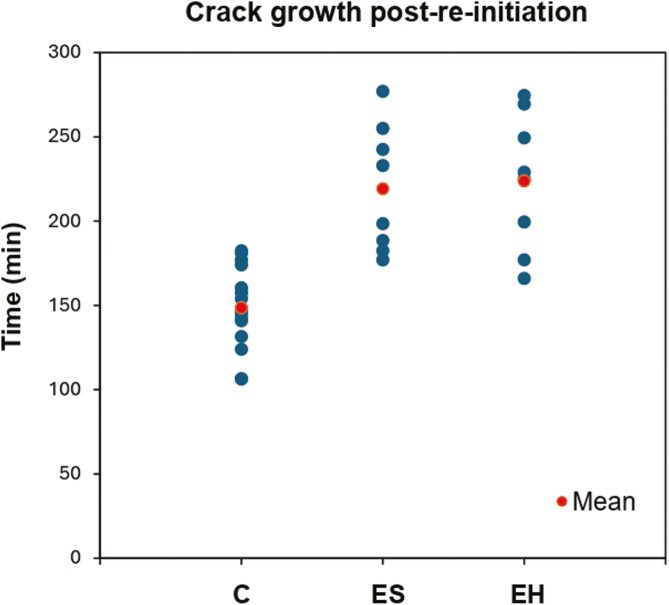
Vertical scatter plot of the post-re-initiation crack growth times across the three groups (min = minutes).

The EH group exhibited the longest mean time of 223.75 ± 40.48 min, closely followed by the ES group with 219.25 ± 37.52 min (Fig. 7). The C group had the lowest mean time of 148.69 ± 23.75 min. Descriptive statistics of the vertical scatter plot are summarized in Table 4. Once again, there was a highly statistically significant difference between the experimental groups and the C group (*P* < .001) (Table 5). There was no statistically significant difference between ES and EH groups (*P* = .821).

**Table 4.** Descriptive statistics of the crack growth post-re-initiation vertical scatter plot. The times should be considered within the context of the 0.5-min resolution of the experiments.

	C—Control	ES—Experimental group #1	EH—Experimental group #2
Mean	148.69	219.25	223.75
Median	148.00	215.75	227
Standard deviation	23.75	37.52	40.48

**Table 5.** Comparisons of the mean post-re-initiation crack growth times using a two independent samples *t*-test.

Two independent samples *t*-test	Findings					
	Sig (*P*-value)	Mean difference	Standard error	95% CI		Result
				Lower	Upper	
C vs ES	<0.001	−70.56	12.49	−96.47	−44.65	Reject null hypothesis
C vs EH	<0.001	−75.06	13.03	−102.08	−48.03	Reject null hypothesis
ES vs EH	0.821	−4.5	19.51	−46.35	37.35	Fail to reject null hypothesis

### Total crack testing (0–10 mm)

**Figure 8.**
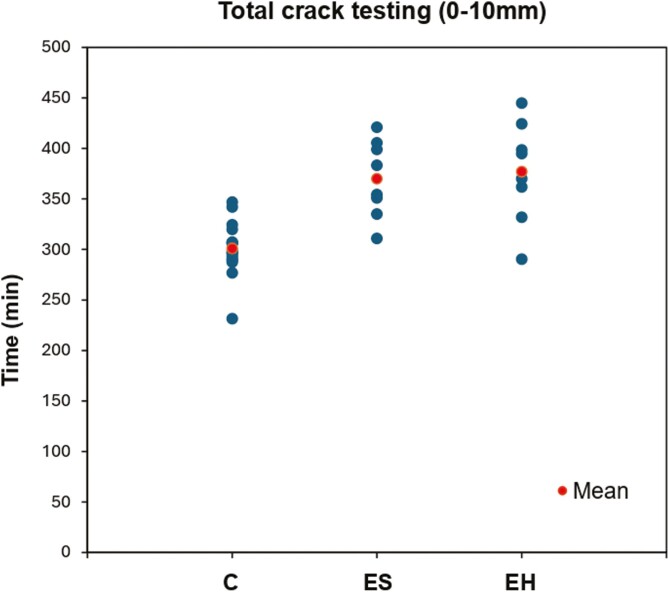
Vertical scatter plot of the total crack testing times across the three groups (min = minutes).

The EH group exhibited the longest mean time of 377.19 ± 49.92 min, closely followed by the ES group with 370.06 ± 38.16 min (Fig. 8). The C group had the lowest mean of 300.97 ± 26.74 min. Descriptive statistics of the vertical scatter plot are summarized in Table 6. There was a highly statistically significant difference in the mean times between the experimental groups and the C group (*P* < .001) (Table 7). There was no statistically significant difference in the mean times between the ES and EH groups (*P* = .248).

**Table 6.** Descriptive statistics of the total crack testing vertical scatter plot. The times should be considered within the context of the 0.5-min resolution of the experiments.

	C—Control	ES—Experimental group #1	EH—Experimental group #2
Mean	300.97	370.06	377.19
Median	297.75	369.00	382.50
Standard deviation	26.74	38.16	49.92

**Table 7.** Comparisons of the mean total crack testing times using a two independent samples *t*-test.

Two independent samples *t*-test	Findings					
	Sig (*P*-value)	Mean difference	Standard error	95% CI		Result
				Lower	Upper	
C vs ES	<0.001	−69.09	13.35	−96.78	−41.40	Reject null hypothesis
C vs EH	<0.001	−76.21	15.49	−108.35	−44.08	Reject null hypothesis
ES vs EH	0.248	−7.13	22.21	−54.77	40.52	Fail to reject null hypothesis

### Overview of the effect of stop-hole placement

**Figure 9.**
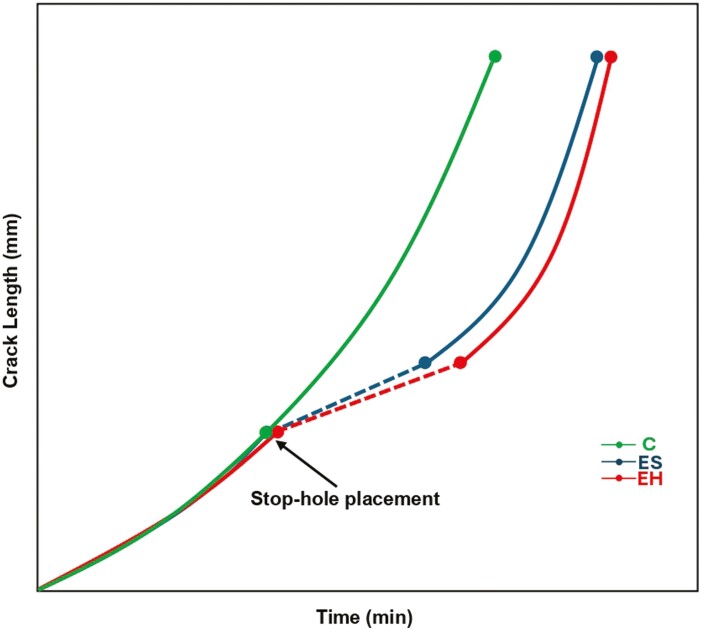
Average effect of stop-hole placement on crack propagation (min = minutes).

An overview of the results is illustrated in a linear graph shown in Fig. 9. Initial crack growth from the starter notch appeared to accelerate exponentially. Immediately after the stop-hole placement, there was a distinct rightward shift in the curve in the ES and EH groups, with an average delay in crack growth of 76 and 98 min, respectively. Continued testing eventually led to the ‘normalization’ of crack growth, where exponential growth was once again observed.

## Discussion

### Fatigue tester

Ensuring an accurate replication of real-life fracture patterns was paramount. To achieve this, several key aspects were implemented. First, the fatigue tester was engineered to reproduce deflection in a TR, inducing crack formation in the incisor region [10, 12]. As previously discussed, the computer simulation of the testing apparatus confirmed the reliability and clinical validity of the setup. Second, the applied load intensity was cyclical and consistently maintained throughout the experiment. Additionally, embedding the TR ends in silicone rubber cushioned and simulated the compliant grip encountered during removal and handling in real-life conditions. Lastly, each TR was positioned within the tester using a custom CAD-CAM-printed retainer clamp and holder, ensuring precise and reproducible placement. Collectively, these measures enhanced the validity of the setup and reliability of the obtained data.

### Crack testing

**Figure 10.**
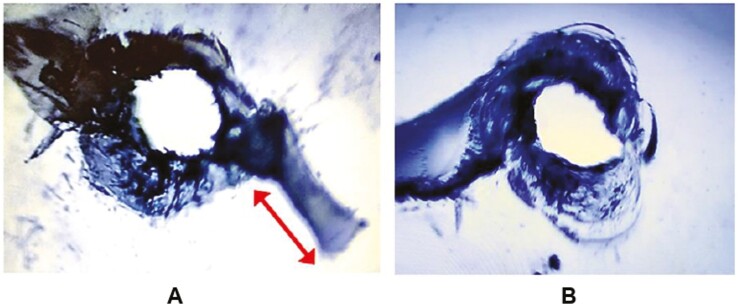
(A) Stop-hole placed with a round carbide bur on a slow-speed handpiece. (B) Stop-hole placed with a heated CPITN probe. Arrow indicates re-initiation of crack from the stop-hole. Both taken under 20× magnification.

There was no statistically significant difference in the overall crack testing times between the ES and EH groups, although the EH group showed a slightly higher re-initiation delay. While this discrepancy might diminish with a larger sample size, its significance did not detract from the overall findings. As anticipated, stop-holes placed with a slow-speed handpiece tended to have rougher margins with micro notches compared to those produced with a heated CPITN probe (Fig. 10). These micro notches may have contributed to the observed difference in the re-initiation times.

Interestingly, the edges of a stop-hole created with a heated CPITN probe exhibited ridge-like lines around its margin (Fig. 10), likely resulting from plastic deformation and material displacement due to heat [21]. This highlights that drilling removes material rather than displacing it. The resulting ridges from the material displacement may add a geometric constraint to the stop-hole, potentially raising local stresses and perversely making crack re-initiation more likely. This sequence of events is consistent with the notion that any imperfection in a material can act as a site of stress concentration [22, 23]. The net result of these two competing mechanisms means that any observed difference between the groups would be subtle, as the data indeed bear out.

It is clear from the linear graph shown in Fig. 9 that the stop-hole is effective at slowing down overall crack propagation. It achieves this by restoring the material to its non-cracked state, requiring a fresh ‘incubation’ period to initiate a new crack. Eventually, continuous stress concentration from testing leads to the re-initiation of crack, reaching a propagation rate comparable to that of the non-stop-holed counterpart. Within the scope of this study, it is estimated that the fatigue life of a TR could be extended by 7500–10 000 cycles, with each cycle representing one removal and re-insertion of a TR under idealized laboratory conditions. This estimate is made notwithstanding potential variations in removal techniques, daily usage, and the human factors involved in the deployment and care of TRs.

### Limitations

The significant scatter in the data is likely attributable to inconsistencies in the temperature of the heated CPITN probe, as well as variations in the speed and torque of the slow-speed handpiece, which may have influenced the shape, size, and smoothness of the stop-holes. Although it remains unclear how each variable precisely influenced the study’s findings, this assumption is reasonable given the material properties of plastics [22, 23]. The large scatter can also be attributed to inherent material inhomogeneities and challenges in consistently placing the stop-holes in identical positions. Such scatter, however, is not unexpected in fatigue studies, given the highly stochastic nature of fatigue, regardless of how standardized the methodology is. Nevertheless, both experimental groups still exhibited a significant increase in fatigue life, which is reassuring and suggests that there may be some promise in the technique of stop-hole placement.

The absence of factors such as saliva, plaque, thermal changes, and other types of co-existing fatigue stresses, which also play a role in crack behaviour in an intra-oral environment, could have influenced the results [24–27]. However, since these limitations were present in both the control and experimental groups, it is hoped that they are unlikely to significantly affect the overall findings.

The decision not to perform a sample size calculation during the design phase arose from uncertainties surrounding the anticipated variance of data and minimal clinically relevant differences, as this was the first study of its kind. Given that sample size calculations are highly sensitive to these parameters, any significant imprecision would have led to an unreliable estimate. Therefore, we opted for a theoretically estimated sample size as a more cautious approach. A post-hoc sample size calculation, based on a significance level of 0.05 and a power of 95%, confirmed that our sample size was appropriate, suggesting a minimum of eight TRs per group (G*Power, version 3.1.9.7).

### Future considerations

The ability to intercept crack progression before it reaches a critical threshold and induces dimensional instability in the TR is essential. Therefore, future studies should focus on identifying this critical threshold and determining the optimal timing for stop-hole placement.

Another consideration arises from the optimistic assumption that patients can accurately detect and place a precisely positioned stop-hole to encompass the entire crack-tip. The challenges inherent in this approach are evident, and therefore, future studies must also explore the development of simple methods and/or devices to facilitate stop-hole placement by members of the general population.

### Clinical implications

The present study demonstrates a practical and relatively straightforward approach to extending the fatigue life of a cracked and failing TR. While the data clearly support the effectiveness of the stop-hole method, the duration of life extension remains uncertain. Further research in clinical settings is warranted to clarify this, although inherent patient-specific variations and confounding factors may complicate the ability to draw robust conclusions. Nevertheless, transitioning from this *in vitro* study to a clinical trial is the logical scientific progression, as real-world data would provide valuable insights.

Despite the limitations of the experimental design, the highly significant results suggest that the stop-hole method could serve as a temporary intervention for cracked but dimensionally stable TRs. This approach may be particularly beneficial for patients with limited access to orthodontic care. However, the amount of ‘time gained’ is difficult to predict, and patients should be advised to seek an orthodontist for a replacement retainer at the earliest opportunity.

## Conclusions

A 1 mm diameter stop-hole placed at the crack-tip of a 3-mm-long crack line in a TR significantly delayed its re-initiation compared to the control group (*P* < .001). While a stop-hole placed with a heated, ball-ended CPITN probe showed a slightly greater effect in delaying re-initiation than one placed with a carbide bur operated on a slow-speed handpiece, the difference was not significant (*P* = .149). As the first study to investigate the stop-hole method for extending the fatigue life of TRs, this research yields promising results and establishes a foundation for further, much-needed investigation in this area of orthodontics.

## Data Availability

The datasets used for the current study are available from the corresponding author upon reasonable request.

## References

[1] Meade MJ , Millett D. Retention protocols and use of vacuum-formed retainers among specialist orthodontists. J Orthod 2013;40:318–25. 10.1179/1465313313Y.0000000066 24297964

[2] Meade MJ , Millett DT. Vacuum-formed retainers: an overview. Dent Update 2015;42:24–34. 10.12968/denu.2015.42.1.24 26062276

[3] Little RM. Stability and relapse of mandibular anterior alignment: University of Washington studies. Semin Orthod 1999;5:191–204. 10.1016/s1073-8746(99)80010-3 10860071

[4] Rinchuse DJ , Miles PG, Sheridan JJ. Orthodontic retention and stability: a clinical perspective. J Clin Orthod 2007;41:125–32. 17473411

[5] Hichens L , Rowland H, Williams A, et al Cost-effectiveness and patient satisfaction: Hawley and vacuum-formed retainers. Eur J Orthod 2007;29:372–8. 10.1093/ejo/cjm039 17702797

[6] Dogramaci EJ , Littlewood SJ. Removable orthodontic retainers: practical considerations. Br Dent J 2021;230:723–30. 10.1038/s41415-021-2893-3 34117427

[7] Rowland H , Hichens L, Williams A, et al The effectiveness of Hawley and vacuum-formed retainers: a single-center randomized controlled trial. Am J Orthod Dentofac Orthop 2007;132:730–7. 10.1016/j.ajodo.2006.06.019 18068589

[8] Thickett E , Power S. A randomized clinical trial of thermoplastic retainer wear. Eur J Orthod 2010;32:1–5. 10.1093/ejo/cjp061 19828592

[9] Moslemzadeh H , Sohrabi A, Rafighi A, et al Comparison of survival time of Hawley and Vacuum-formed retainers in orthhodontic patients—a randomized clinical trial. Adv Biosci Clin Med 2017;5:7. 10.7575/aiac.abcmed.17.05.01.02

[10] Sun J , Yu YC, Liu MY, et al Survival time comparison between Hawley and clear overlay retainers: a randomized trial. J Dent Res 2011;90:1197–201. 10.1177/0022034511415274 21771797

[11] Jin C , Bennani F, Gray A, et al Survival analysis of orthodontic retainers. Eur J Orthod 2018;40:531–6. 10.1093/ejo/cjx100 29370399

[12] Zhu Y , Lin J, Long H, et al Comparison of survival time and comfort between 2 clear overlay retainers with different thicknesses: a pilot randomized controlled trial. Am J Orthod Dentofac Orthop 2017;151:433–9. 10.1016/j.ajodo.2016.10.019 28257727

[13] Doğramacı E , Chubb D, Rossi-Fedele G. Orthodontic thermoformed retainers. A two‐arm laboratory study into post‐fabrication outcomes. Aust Dent J 2018;63:347–55. 10.1111/adj.12610 29660138

[14] Mizuhashi F , Koide K. Appropriate fabrication method for vacuum-formed mouthguards. Dent Traumatol 2017;33 110–3. 10.1111/edt.12302 27500893

[15] Mizuhashi F , Koide K, Mizuhashi R. Influence of working model angle on the formation of a pressure-formed mouthguard. Dent Traumatol 2017;33:189–93. 10.1111/edt.12317 27960037

[16] Song PS , Shieh YL. Stop drilling procedure for fatigue life improvement. Int J Fatigue 2004;26:1333–9. 10.1016/j.ijfatigue.2004.04.009

[17] Wang F , Wang M. Effect of holes on dynamic crack propagation under impact loading. Appl Sci 2020;10:1122. 10.3390/app10031122

[18] Ayatollahi MR , Razavi SMJ, Sommitsch C, et al Fatigue life extension by crack repair using double stop-hole technique. Mater Sci Forum 2016;879:3–8.

[19] Schiøtz J , Carlsson AE, Canel LM, et al Effect of crack blunting on subsequent crack propagation. MRS Online Proceedings Library 1995;409:237–42. 10.1557/PROC-408-237

[20] Goh SY. Post-thermoforming thickness and fatigue testing of thermoplastic retainers. Master’s dissertation, University College London, 2021, 1–83.

[21] Whitley K , Gates T. Thermal/mechanical response of a polymer matrix composite at cryogenic temperatures. AIAA J 2003;42:1–13. 10.2514/1.1063

[22] Crawford RJ. Chapter 2—Mechanical behaviour of plastics. In: Crawford RJ (ed.), Plastics Engineering, 3rd edn. Oxford: Butterworth-Heinemann, 1998, 41–167.

[23] Crawford RJ. Chapter 1—General properties of plastics. In: Crawford RJ (ed.), Plastics Engineering, 3rd edn. Oxford: Butterworth-Heinemann, 1998, 1–40.

[24] Cianci C , Pappalettera G, Renna G, et al Mechanical behavior of PET-G tooth aligners under cyclic loading. Front Mater 2020;7:104. 10.3389/fmats.2020.00104

[25] Fang D , Zhang N, Chen H, et al Dynamic stress relaxation of orthodontic thermoplastic materials in a simulated oral environment. Dent Mater J 2013;32:946–51. 10.4012/dmj.2013-131 24240895

[26] Kwon JS , Lee YK, Lim BS, et al Force delivery properties of thermoplastic orthodontic materials. Am J Orthod Dentofac Orthop 2008;133:228–34; quiz 328.e1. 10.1016/j.ajodo.2006.03.034 18249289

[27] Ryokawa H , Miyazaki Y, Fujishima A, et al The mechanical properties of dental thermoplastic materials in a simulated intraoral environment. Orthod Waves 2006;65:64–72. 10.1016/j.odw.2006.03.003

